# Factors Influencing the Differentiation of Human Monocytic Myeloid-Derived Suppressor Cells Into Inflammatory Macrophages

**DOI:** 10.3389/fimmu.2018.00608

**Published:** 2018-03-26

**Authors:** Defne Bayik, Debra Tross, Dennis M. Klinman

**Affiliations:** Cancer and Inflammation Program, National Cancer Institute at Frederick, Frederick, MD, United States

**Keywords:** myeloid-derived suppressor cells, inflammatory macrophage, TNFα, STAT4, NF-κB, IFNγ

## Abstract

Monocytic myeloid-derived suppressor cells (mMDSC) accumulate within tumors where they create an immunosuppressive milieu that inhibits the activity of cytotoxic T and NK cells thereby allowing cancers to evade immune elimination. The toll-like receptors 7/8 agonist R848 induces human mMDSC to mature into inflammatory macrophage (MAC_inflam_). This work demonstrates that TNFα, IL-6, and IL-10 produced by maturing mMDSC are critical to the generation of MAC_inflam_. Neutralizing any one of these cytokines significantly inhibits R848-dependent mMDSC differentiation. mMDSC cultured in pro-inflammatory cytokine IFNγ or the combination of TNFα plus IL-6 differentiate into MAC_inflam_ more efficiently than those treated with R848. These mMDSC-derived macrophages exert anti-tumor activity by killing cancer cells. RNA-Seq analysis of the genes expressed when mMDSC differentiate into MAC_inflam_ indicates that TNFα and the transcription factors NF-κB and STAT4 are major hubs regulating this process. These findings support the clinical evaluation of R848, IFNγ, and/or TNFα plus IL-6 for intratumoral therapy of established cancers.

## Introduction

Infiltration of the tumor microenvironment by immunosuppressive leukocytes protects cancers from immune elimination (1). Myeloid-derived suppressor cells (MDSC) are key contributors to this immunosuppressive milieu. MDSC are classified into monocytic or granulocytic subsets based on their phenotype, morphology, and function. Both subsets are present at very low frequencies in the peripheral blood of healthy donors but are much more prevalent in cancer patients (2–5). MDSC migrate from the peripheral blood into the tumor bed, where they inhibit the activity of tumoricidal NK and cytotoxic T cells (5, 6). As monocytic myeloid-derived suppressor cells (mMDSC) have the greatest immunosuppressive activity per cell, efforts to enhance the efficacy of immunotherapy have focused on blocking the recruitment/activation of that cell type (2, 4, 5).

Toll-like receptors (TLRs) comprise a family of highly conserved germline-encoded pattern recognition receptors (7). TLR engagement stimulates elements of the innate immune system to produce pro-inflammatory cytokines, such as IL-6 and IL-12 that help bolster adaptive immunity (7, 8). Our group previously showed that injecting TLR7 agonists into murine tumors induced resident mMDSC to differentiate into tumoricidal M1-like macrophages (MAC_inflam_) and led to the elimination of established cancers (9, 10). Human mMDSC cultured with the TLR7/8 agonist R848 also differentiate primarily into MAC_inflam_ (11). By comparison, human mMDSC treated with the TLR2/1 agonist Pam3CSK4 (hereafter PAM3) mature primarily into immunosuppressive M2-like macrophages (MAC_suppress_) (11). This study seeks to identify the factors and gene networks that influence the generation of MAC_inflam_ from mMDSC by comparing the effects of R848 treatment with that of other stimulants.

## Materials and Methods

### Reagents

R848, Pam3CSK4, Celastrol, and Ruxolitinib were purchased from InvivoGen (San Diego, CA, USA) and all human recombinant cytokines were obtained from Miltenyi Biotec (Auburn, CA, USA). CD163 (Clone #GHI/61), CD206 (Clone #15-2), and CD14 (Clone #M5E2) antibodies used to purify or stain human mMDSC, and anti-IL-6 (Clone #MQ2-13A5), anti-IL-10 (Clone #JES3-19F1), anti-IL-12 (Clone #C11.5), anti-TNFα (Clone #Mab1), and anti-IFNγ (Clone #NIB42) utilized to neutralize secreted cytokines were purchased from BioLegend (San Diego, CA, USA) with the exception of CD14 (Clone #MφP9), EGFR (Clone #EGFR.1), and HLA-DR (Clone #G46-6) antibodies (BD Biosciences, Franklin Lakes, NJ, USA) and the marker of active macrophage 25F9 (Clone #eBio25F9, Thermo Scientific, Waltham, MA, USA).

### Preparation of Human mMDSC

Elutriated mononuclear cells and apheresis collections were obtained from healthy donors on NCI IRB-approved NIH protocol 99-CC-0168. Research blood donors provided written informed consent and blood samples were de-identified prior to distribution (NCT00001846) (National Institutes of Health, Bethesda, MD, USA). Peripheral blood mononuclear cells (PBMC) and elutriated monocytes were separated by gradient centrifugation over Histopaque (Sigma-Aldrich, St. Louis, MO, USA) and cultured overnight in RPMI 1640 medium supplemented with 2% FCS (both from Lonza, Walkersville, MD, USA), 2 mM glutamine, and 25 mM HEPES buffer (both from Invitrogen, Carlsbad, CA, USA). Elutriated mononuclear cells or PBMC in suspension were stained with fluorescence-conjugated antibodies against CD14 and HLA-DR. mMDSC represented by CD14^bright^ HLA-DR^−/low^ population was FACS sorted using a FACSAria II (BD Biosciences, Franklin Lakes, NJ, USA) with >95% purity.

### *In Vitro* Stimulation of mMDSC

FACS-purified mMDSC were stimulated with 1 µg/ml PAM3, 3 µg/ml R848 [previously defined to be the optimal concentration to drive mMDSC maturation (11)], and/or 250 ng/ml of IL-6, IL-10, IL-12, TNFα, IFNγ, or M-CSF in RPMI supplemented with 2% FCS. Where indicated, cytokine neutralizing Abs (25 µg/ml), the IκB kinase (IKK) inhibitor Celastrol (1 µM), and/or the Janus kinase1/2 (JAK1/2) inhibitor Ruxolitinib (1 µM) were added throughout the duration of MDSC culture (3–5 days).

### Analysis of Surface Marker Expression by mMDSC

Stimulated mMDSC were incubated with Fc Block for 15 min on ice and stained with fluorochrome-conjugated antibodies against 25F9, CD206, and CD163 on ice for 20 min. Cells were washed with PBS/2% BSA followed by Fix & Perm Medium A (Invitrogen, Carlsbad, CA, USA). Cells were washed again, re-suspended in PBS, and analyzed using an LSRFortessa (BD Biosciences, Franklin Lakes, NJ, USA).

### Cytotoxicity Assay

FACS-purified mMDSC were cultured with R848, IL-6 plus TNFα, M-CSF, or IFNγ for 5 days. Cells were then collected through scraping, counted, and incubated with A549 tumor cells at a 1:40 ratio in fresh media for 6 h. Samples were trypsinized and stained with LIVE/DEAD Fixable Near-IR Dead Cell Stain Kit (Life Technologies, Carlsbad, CA, USA) followed by fluorescein-conjugated anti-EGFR and anti-CD14 Ab for 30° on ice. After washing, cells were re-suspended in PBS/2% BSA and analyzed using the LSRFortessa.

### ELISAs

Cell supernatants were collected on day 3 and frozen until further use. Immunol 2HB microtiter plates (Thermo Scientific) were coated with anti-cytokine antibodies anti-IL-6 (Clone #6708), anti-IL-10 (Clone #127107), anti-TNFα (Clone #28401), and anti-M-CSF (Clone #21113) (R&D Systems, Minneapolis, MN, USA) and then blocked with PBS/2% BSA. Serially diluted standards and culture supernatants were added to these plates overnight. Plates were incubated with biotinylated anti-cytokine Ab (R&D Systems), followed by phosphatase-streptavidin (BD Biosciences) and K-Gold PNPP Substrate (Neogen Corporation, Lexington, KY, USA). Human IL-12p70 Quantikine, IL-4 Quantikine, and TGFβ1 Quantikine ELISAs were performed based on manufacturer’s instructions (R&D Systems). ELISAs were read using a SpectraMax M5 Microplate Reader and SoftMax Pro Acquisition and Analysis Software (both Molecular Devices, Sunnyvale, CA, USA).

### RNA-Seq Analysis

After 4 h stimulation [a duration previously found to be optimal for monitoring changes in gene expression in differentiating mMDSC (11)], stimulated mMDSC were stored in RNA Protect (Qiagen, Frederick, MD, USA). Total RNA was isolated using the RNeasy micro kit (Qiagen) and RNA quality was assessed using an Agilent 2200 TapeStation. mRNA libraries were generated using the Smart-Seq ultra-low input kit (Clontech) and sequenced using a HiSeq2500 sequencer using IlluminaTruSeq v4 chemistry with 125 bp paired-end reads. Sequences were aligned to the human (hg19) reference genome. Genes that were differentially expressed compared to untreated samples were identified using CLC genomics workbench (version 10). Genes that were significantly upregulated (FDR *p* < 0.01) were imported into Ingenuity Pathway Analysis (Qiagen, version 10). Networks involving genes that interacted with more than two other genes were used to build networks. Accession code in GEO repository: GSE105142.

### Statistical Analysis

A two-sided unpaired Student’s *t*-test was used for analysis, and *p*-value less than 0.05 was considered statistically significant (GraphPad Software Inc., La Jolla, CA, USA).

## Results

### R848 Induces mMDSC to Differentiate Into MAC_inflam_

**Figure 1:**
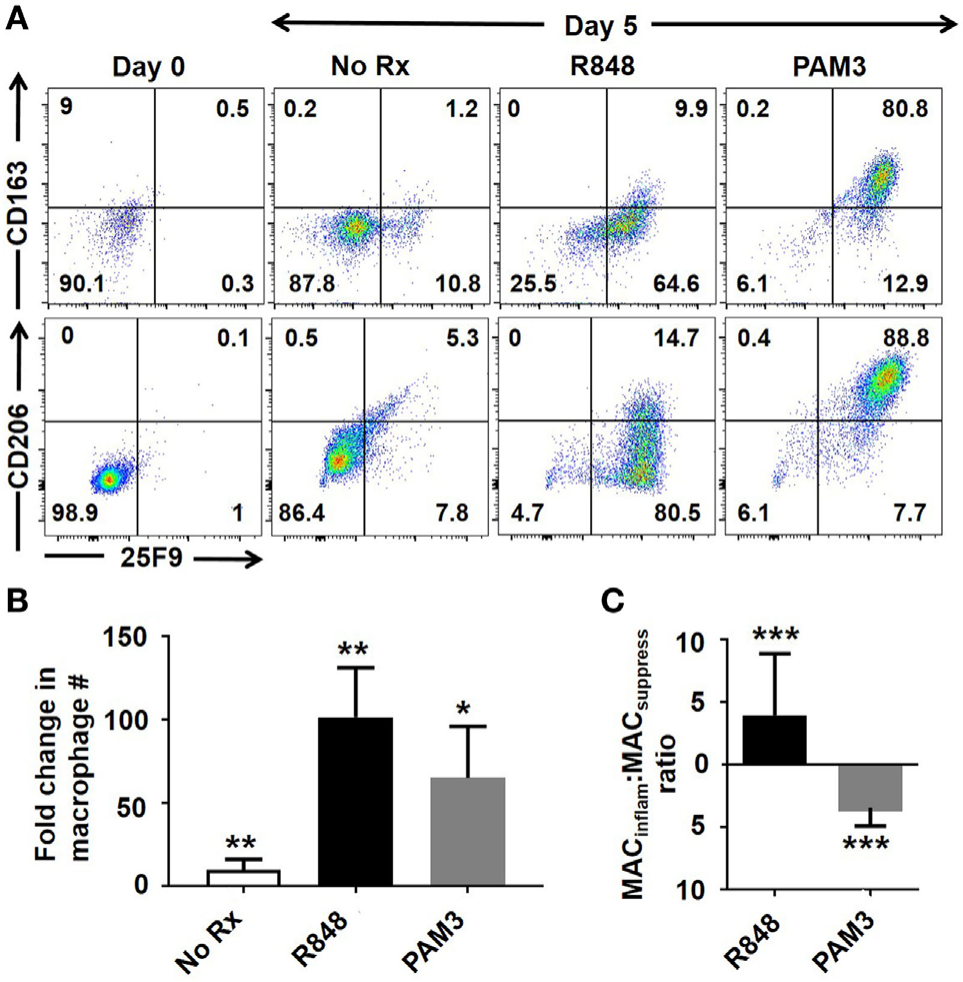
Effect of Toll-like receptor stimulation on mMDSC polarization. FACS-purified human mMDSC were stimulated with R848 (3 µg/ml) or PAM3 (1 µg/ml) for 5 days. **(A)** Representative dot plots depicting changes in 25F9, CD163, and CD206 expression. **(B)** Fold change in the number of macrophage present at the end of culture over the baseline of freshly isolated mMDSC (mean ± SD of six independently studied donors/data point). **(C)** Ratio of CD163^+^ to CD163^−^ 25F9^+^ macrophage in the samples described in **(B)**. *p < 0.05, **p < 0.01, ***p < 0.001 versus unstimulated cells.

Macrophage has historically been categorized into two subsets: MAC_inflam_ and MAC_suppress_ (12). These subsets differ both phenotypically and functionally. While all human macrophages express the 25F9 surface marker, only MAC_suppress_ upregulate the CD163 scavenger receptor and the CD206 C-type mannose receptor (13, 14). Our lab previously demonstrated that the TLR7/8 agonist R848 induced human mMDSC to differentiate into MAC_inflam_, while the TLR2/1 agonist PAM3 supported their preferential generation of MAC_suppress_ (11). To clarify the mechanism underlying the generation of MAC_inflam_, normal healthy volunteers were leukapheresed and mMDSC were isolated by FACS sorting based on the absence of HLA-DR and presence of CD14 (3, 11). As previously documented, mMDSC constitute 0.4 + 0.3% of PBMC in normal donors (11). Consistent with the earlier report, TLR stimulation induced a majority of CD14^+^/HLA-DR^−^ mMDSC to differentiate into 25F9^+^ macrophage (Figures 1A,B). Preliminary studies further showed that increasing the duration of culture from 3 to 5 days increased the generation of MAC_inflam_ following R848 stimulation without altering the generation of MAC_suppress_ by PAM3 (Figure S1 in Supplementary Material, *p* < 0.05). In the absence of stimulation, less than 1% of mMDSC survived 5 days in culture (yielding too few cells for further study). By contrast, viability was high in cultures stimulated with R848 or PAM3 (80.2 + 11.3%).

A majority of the macrophage generated by R848 treatment expressed only 25F9 and thus were phenotypically MAC_inflam_ (Figure 1C). This contrasted to PAM3 treated cells that typically expressed CD163 and CD206 in addition 25F9 (Figures 1A,C). On average, R848 treatment generated a four-fold excess of MAC_inflam_ compared to MAC_suppress_, while PAM3 generated fourfold more MAC_suppress_ than MAC_inflam_ (Figure 1C), consistent with previous findings (11).

### Contribution of Cytokines to R848-Induced Generation of MAC_inflam_

**Figure 2:**
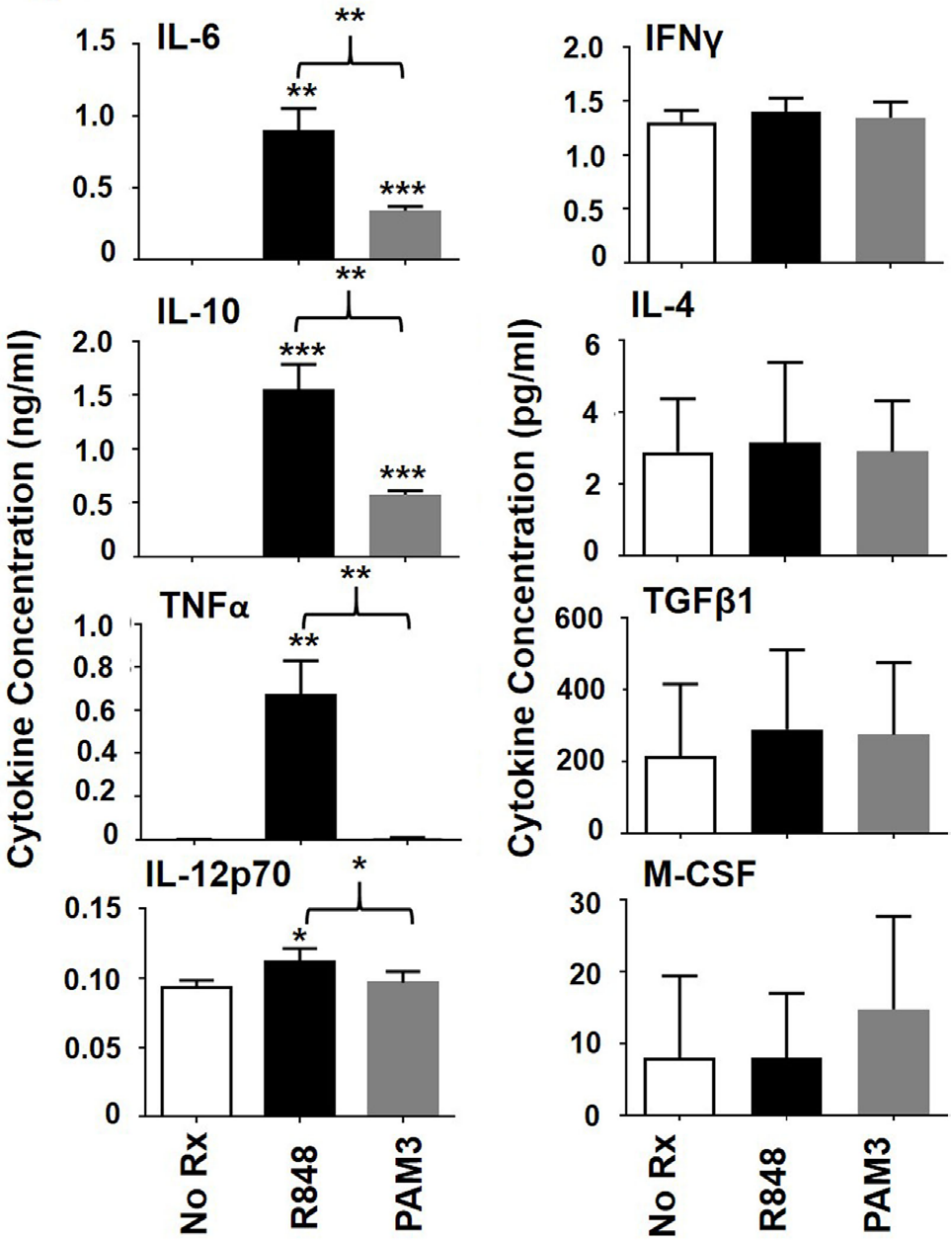
Effect of Toll-like receptor stimulation on cytokine production by mMDSC. FACS-purified mMDSC were stimulated as described in Figure 1. Cytokine and chemokine levels in culture supernatants were determined on day 3 by ELISA (mean ± SD of samples from four to eight independently analyzed donors). *p < 0.05; **p < 0.01, ***p < 0.001 versus unstimulated cells or between PAM3 and R848 stimulated mMDSC.

To better understand the factors that influence the generation of MAC_inflam_ rather than MAC_suppress_, the production of cytokines by human mMDSC stimulated with R848 versus PAM3 was compared. Our lab previously used intracytoplasmic cytokine staining to show that mMDSC cultured for 1–3 days with R848 accumulated cells containing IL-6 and IL-12, while those treated with PAM3 accumulated cells containing IL-6 and IL-10 (11). To measure secreted cytokine levels in the surrounding environment, culture supernatants (representing the balance of cytokines produced and metabolized over 3 days) were examined. Levels of IL-6 and IL-10 rose significantly after stimulation with either R848 or PAM3, with concentrations being higher after R848 stimulation (Figure 2). By contrast, the pro-inflammatory cytokines TNFα and IL-12 were significantly elevated only after R848 treatment (Figure 2). Levels of IFNγ, M-CSF, IL-4, and TGFβ1 did not change in response to either TLR agonist. These results suggested that TNFα and/or IL-12 might contribute to the preferential generation of MAC_inflam_ mediated by R848.

**Figure 3:**
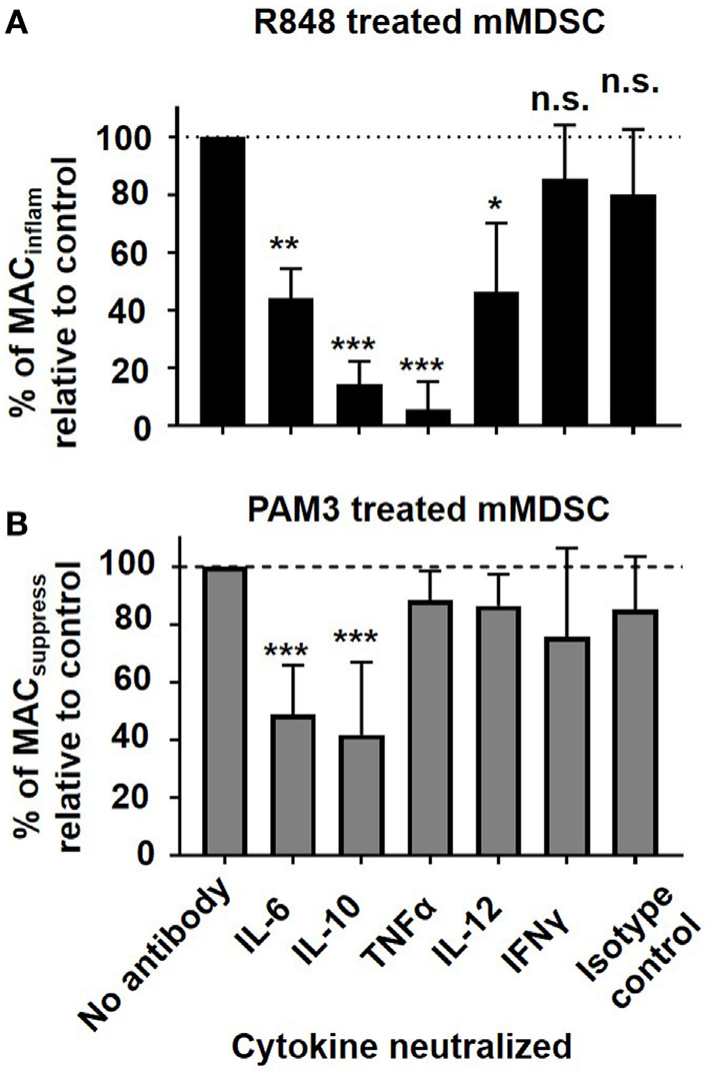
Effect of cytokine neutralization on Toll-like receptor-induced macrophage generation. FACS-purified mMDSC were stimulated with R848 or PAM3 for 3 days in the presence of 25 µg/ml neutralizing anti-cytokine or isotype control antibodies. Data show the change in frequency of **(A)** MAC_inflam_ generated by R848 versus **(B)** MAC_suppress_ generated by PAM3 (mean ± SD of samples from 4 to 11 independently analyzed donors/group). *p < 0.05; **p < 0.01, ***p < 0.001 versus isotype control antibody group.

Cytokine neutralization experiments were conducted to examine these possibilities. Blocking TNFα resulted in a dramatic reduction in the generation of MAC_inflam_ but had no effect on the number of MAC_suppress_. This finding suggests that TNFα may play an important role in determining the type of macrophage generated following TLR stimulation of mMDSC (Figure 3). Blocking IL-6 or IL-10 reduced the generation of both MAC_suppress_ and MAC_inflam_ (*p* < 0.01), consistent with those cytokines contributing to the general process by which mMDSC mature into macrophage (Figure 3). Blocking IL-12 led to a modest reduction in MAC_inflam_, while having no effect on MAC_suppress_. The addition of neutralizing Ab against cytokines that were not detected in stimulated cultures (such as IFNγ) had no effect on macrophage generation (Figure 3).

### Cytokines Can Directly Induce mMDSC Differentiation

**Figure 4:**
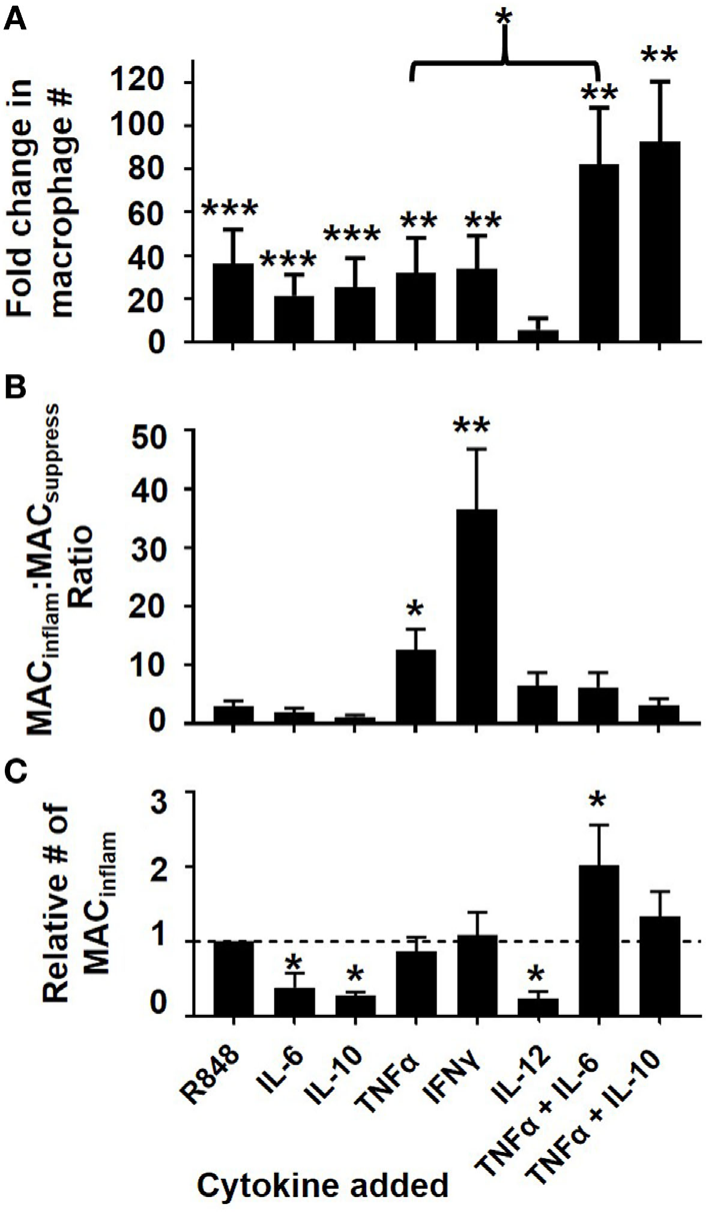
Effect of cytokines on mMDSC polarization. FACS-purified mMDSC were stimulated with 3 µg/ml of R848 or various cytokines (250 ng/ml) for 5 days. **(A)** Fold change in the fraction of macrophage present at the end of culture (mean ± SD of five to eight independently studied donors/data point), **(B)** ratio of CD163^+^ to CD163^−^ macrophage present at the end of culture, **(C)** relative number of MAC_inflam_ present in culture versus a baseline of R848 for each donor. *p < 0.05, **p < 0.01, ***p < 0.001 versus unstimulated cells.

To further examine whether TNFα and to a lesser extent IL-6, IL-10, and/or IL-12 could influence the differentiation of mMDSC, their effect was compared to that of R848. The addition of IL-6 and/or IL-10 significantly increased the generation of macrophages from mMDSC but did not preferentially support the generation of MAC_inflam_ (Figure 4). By comparison, TNFα mirrored the ability of R848 to generate MAC_inflam_ rather than MAC_suppress_ (Figure 4B). IL-12 had no significant effect on the maturation of mMDSC into MAC_inflam_ (Figures 4A,C).

Based on the observation that IL-6 and IL-10 supported the general process of mMDSC differentiation, the effect of co-administering them with TNFα was examined. The combination of TNFα plus IL-6 generated a greater number of MAC_inflam_ than any other treatment (*p* < 0.05; Figure 4C). IFNγ was also evaluated in these studies. While IFNγ played no role in R848-driven mMDSC maturation (Figures 2 and 3), previous reports indicated that IFNγ could induce classical monocytes to differentiate into MAC_inflam_ (15, 16). Current results show that IFNγ also supports the generation of MAC_inflam_ from mMDSC (Figure 4). These findings suggest that multiple distinct stimuli can play a role in the generation of MAC_inflam_. To examine that possibility, subsequent experiments focusing on the mechanism underlying the generation of 25F9^+^CD163^−^ macrophage compared the effect of TNFα plus IL-6 to IFNγ as well as to R848.

### Functional Activity of MAC_inflam_ Generated From mMDSC

**Figure 5:**
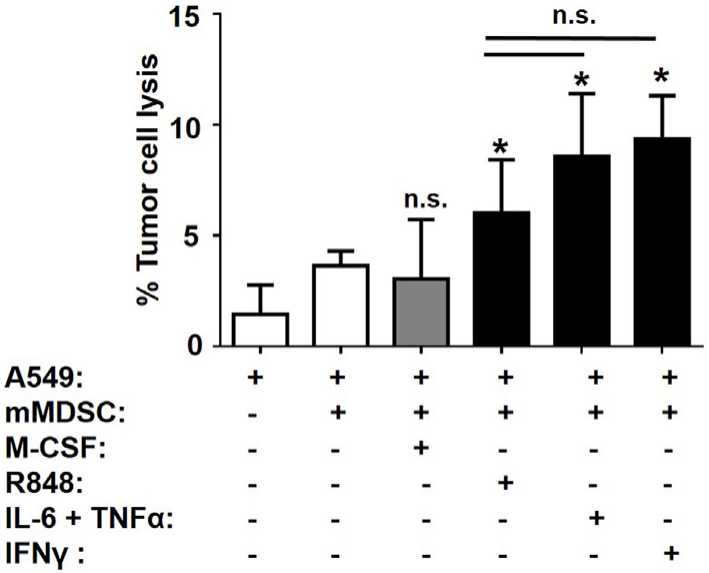
Tumoricidal activity of MAC_inflam_. Purified mMDSC were stimulated with 3 µg/ml R848 or 250 ng/ml IL-6, TNFα, IFNγ, and/or M-CSF for 5 days. Their ability to lyse A549 tumor targets during 6 h of incubation at an E:T ratio of 40:1 is shown. Data shown mean ± SD of four to eight independently analyzed donors per group. *p < 0.05 versus unstimulated mMDSC.

We previously established that MAC_inflam_ but not MAC_suppress_ could lyse A549 tumor cells. The functional activity of MAC_inflam_ generated by treating mMDSC with R848, IL-6 plus TNFα, or IFNγ was therefore evaluated using this assay. As expected, MAC_inflam_ generated by R848 treatment lysed tumor targets (*p* < 0.05; Figure 5). MAC_inflam_ produced in cultures containing IL-6 plus TNFα or IFNγ also mediated significant tumor cell lysis (Figure 5). There was no statistically significant difference in the activity of MAC_inflam_ generated by any of these treatments, suggesting the MAC_inflam_ generated by distinct stimuli were not only phenotypically alike but also shared functional characteristics.

### Regulatory Networks Underlying the Differentiation of mMDSC Into MAC_inflam_

**Figure 6:**
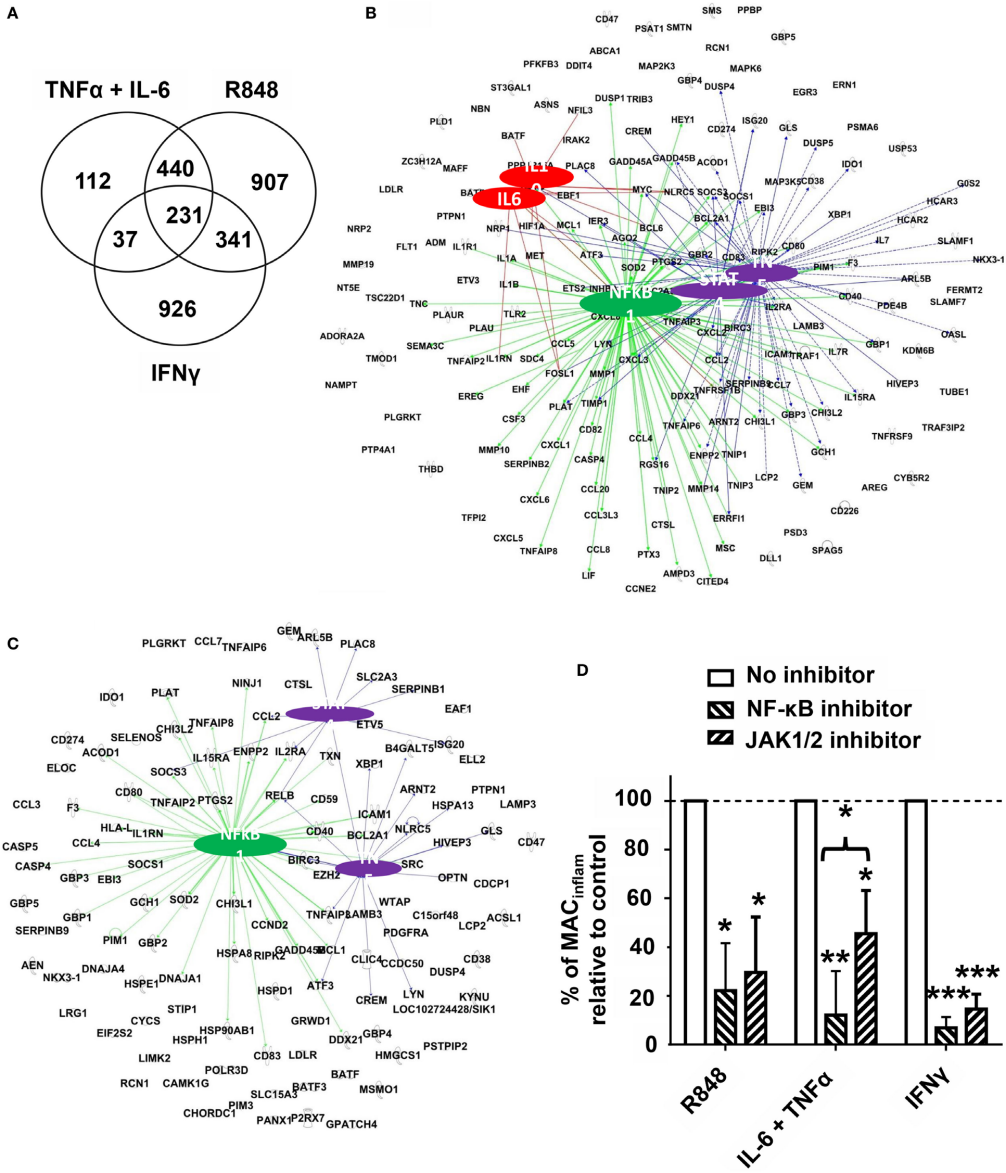
Regulatory networks associated with differentiation of mMDSC into MAC_inflam_. Purified mMDSC were stimulated with R848, IFNγ, or the combination of TNFα plus IL-6 for 4 h. Changes in gene expression were detected using RNA-Seq by comparison to unstimulated samples from the same donors. **(A)** Venn diagram showing the number of genes upregulated by each treatment. **(B)** Genes activated by both R848 and IL-6 plus TNFα whose regulatory interactions could be mapped by IPA. Colored lines identify regulatory gene interactions involving NF-κB (green), TNFα or STAT4 (purple), and IL-6 or IL-10 (red). **(C)** Genes activated by all three methods of MAC_inflam_ generation (R848, IFNγ, and TNFα plus IL-6). Colored lines identify regulatory gene interactions involving NF-κB (green) and TNFα or STAT4 (purple). **(D)** Purified mMDSC were stimulated with R848, IFNγ, or TNFα plus IL-6 for 5 days in the continuous presence of the IκB kinase inhibitor Celastrol or the Janus kinase1/2 (JAK1/2) inhibitor Ruxolitinib (mean ± SD of three to four independently studied donors/data point). Inhibitors were added on day 0. *p < 0.05, **p < 0.01 versus unstimulated cells.

The above findings established that mMDSC treated with R848, IFNγ, or the combination of TNFα plus IL-6 matured into MAC_inflam_ based on both phenotypic and functional metrics. Previous studies examined the gene expression signatures of mMDSC stimulated with PAM3 versus R848 (9). While differences in mRNA levels were detected and the optimal timepoint for analyzing shifts in gene expression identified (4 h) that study was unable to identify the regulatory networks underlying the generation of MAC_inflam_ (11). We reasoned that comparing the effects of R848, IFNγ, and TNFα plus IL-6 treatments might clarify whether there was a common pathway underlying the differentiation of mMDSC into MAC_inflam_. To test that possibility, mRNA libraries generated from mMDSC cultured for 4 h with each stimulant were sequenced. Significantly upregulated genes (*p* < 0.01) that formed network connections with at least two other genes were identified. TNFα plus IL-6 upregulated 820 genes, whereas R848 upregulated ~2.3 times that many (Figure 6A). Consistent with the observation that blocking either IL-6 or TNFα significantly inhibited R848-driven mMDSC differentiation, 82% of the genes upregulated by IL-6 plus TNFα were also upregulated by R848 (Figure 6A). By contrast, only 37% of the genes upregulated by IFNγ were shared with R848 (Figure 6A).

Ingenuity Pathway Analysis was used to identify the regulatory networks involved in the activation of these shared genes. Initial studies compared R848 with IL-6 plus TNFα, given the close relationship already established between those two forms of mMDSC activation. Consistent with results from the cytokine neutralization studies (Figure 3), TNFα and to a lesser extent IL-6 and IL-10 were found to be regulatory hubs associated with the maturation of mMDSC into MAC_inflam_ (Figure 6). TNFα influenced the expression of a large set of genes associated with the generation of MAC_inflam_ that was largely distinct from those regulated by IL-6 or IL-10 (Figure 6B). IPA also predicted that regulatory networks mediated *via* the NF-κB complex and STAT4 would be relevant to the generation of MAC_inflam_ (Figure 6B). To determine whether the same regulatory pathways contributed to the generation of MAC_inflam_ mediated by an unrelated stimulant, we performed IPA analysis of the genes upregulated by IFNγ as well R848 and IL-6 plus TNFα. This analysis confirmed that NF-κB, STAT4, and TNFα were major hubs regulating the differentiation of mMDSC into MAC_inflam_ (Figure 6C). Of interest, mMDSC stimulated with IFNγ did not upregulate expression of IL-6/IL-10 but instead triggered genes encoding IFNγ-induced regulatory factors (Figure 6C and data not shown).

To validate the IPA prediction that the transcription factors NF-κB and STAT4 drove MAC_inflam_ differentiation and inhibitors of IKK (an upstream regulator of the NF-κB complex) and JAK1/2 (an upstream regulator of STAT4) were added throughout the period of mMDSC culture. Inhibition of either the NF-κB complex or JAK-STAT4 axis blocked the polarizing activity of R848, IFNγ, and IL-6 plus TNFα by 55–90%, consistent with the conclusion that these pathways are essential for the generation of MAC_inflam_ from mMDSC (Figure 6D).

## Discussion

Myeloid-derived suppressor cells contribute to the immunosuppressive microenvironment that protects cancers from elimination by tumoricidal NK and T cells (5). In animal models, survival is significantly prolonged by interventions that block the generation, expansion, and/or trafficking of MDSC to the tumor bed (17–19). Epidemiologic studies show that the presence of large numbers of MDSC correlates with a worse prognosis and poorer response to therapy in cancer patients (20–22). Targeting MDSC has thus become an important component of cancer immunotherapy as they are highly immunosuppressive and accumulate in the tumor microenvironment (2, 4, 5, 19, 23). Clinical trials designed to eliminate mMDSC and/or induce them to differentiation into tumoricidal macrophages are underway (17–19). In support of that goal, this work examined the mechanisms regulating the maturation of human mMDSC into MAC_inflam_.

Previous studies showed that TLR7/8 agonists, including R848, preferentially induced mMDSC isolated from healthy donors or cancer patients to mature into tumoricidal macrophage with the ability to produce pro-inflammatory cytokines (11). Studies in mice verified that intratumoral delivery of TLR7 agonists facilitated the elimination of large tumors, an effect accompanied by the differentiation of mMDSC into MAC_inflam_ (9, 10, 24). Yet, not all TLR agonists have this effect on human mMDSCs: the TLR2/1 agonist PAM3 induces them to differentiate into MAC_suppress_ that interfere with tumor elimination (11). Yet mMDSC differentiation has a stochastic component in that a minority of the macrophage generated by R848 were suppressive, while a minority of those generated by PAM3 were inflammatory (Figure 1) (11). This work sought to clarify the processes that support the generation of MAC_inflam_ with the goal of improving their generation for anti-tumor therapy.

As previously reported, (1) R848 preferentially supported the generation of 25F9^+^CD163^−^ MAC_inflam_, while PAM3 supported the generation primarily of 25F9^+^CD163^+^ MAC_suppress_ (Figure 1) and (2) R848 elicited the production of a different pattern of cytokines than PAM3 (Figure 2) (11). Expanding on those findings, this work examined additional cytokines and found that TNFα production was significantly elevated in R848 but not PAM3 stimulated cultures (Figure 2). Given the limited number of cytokines detected in R848-treated mMDSC cultures, we explored whether they might play a role in the generation of MAC_inflam_. Consistent with that possibility, neutralizing TNFα (and to a much lesser extent IL-12) significantly reduced the generation of MAC_inflam_ by R848, while leaving the generation of MAC_suppress_ by PAM3 intact (Figure 3). Neutralizing IL-6 or IL-10 blocked the development of both MAC_inflam_ and MAC_suppress_, indicating that those cytokines contribute to the general process of mMDSC maturation. TNFα, IL-6, and IL-10 (but not IL-12) also supported the differentiation of freshly isolated mMDSC into macrophages yet only TNFα selectively supported the generation of MAC_inflam_ (Figure 4). Of interest, although IFNγ was not present in TLR-stimulated cultures and had no effect on R848-dependent generation of MAC_inflam_, that cytokine was able to stimulate human mMDSC to differentiate into MAC_inflam_. This finding builds on earlier studies showing that IFNγ supports the generation of antigen presenting cells from classical monocytes (15, 16).

Several earlier studies examined the effect of TNFα and IFNγ on murine rather than human MDSC. While the accumulation of MDSC in inflammatory states was initially associated with increased TNFα and IL-6 levels (25–28), more recent data suggest that increased TNFα expression in the tumor microenvironment reduces MDSC infiltration and supports tumor regression (29). Similarly, IFNγ was shown to augment the suppressive activity of murine MDSC by triggering nitric oxide production, a mediator used by MDSCs to suppress T cell activity (30, 31). However, human myeloid cells differ from mice in terms of their ability to produce inducible nitric oxide synthase and respond to some stimulants (32). Given the importance of mMDSC in tumor immunology, this work examined how the differentiation of these cells was regulated in humans. As seen in Figure 4, our findings indicate that TNFα and IFNγ induce human mMDSC to differentiate into MAC_inflam_.

Consistent with the cytokine neutralization data, adding IL-6 or IL-10 to cultures of human mMDSC increased total macrophage yield but did not selectively generate MAC_inflam_ rather than MAC_suppress_ (Figures 4A,B). By contrast, TNFα and IFNγ induced mMDSC to preferentially differentiate into MAC_inflam_. The yield and relative frequency of MAC_inflam_ was maximized by treating mMDSC with a combination of TNFα plus IL-6 (Figure 4C). Coupled with the observation that neutralizing TNFα or IL-6 significantly inhibited the activity of R848 (Figure 3), these findings identify TNFα as a central driver of R848-induced mMDSC polarization and suggest that the general process by which mMDSC differentiate into macrophage is supported by IL-6 and perhaps IL-10. Inflammatory macrophages contribute to cancer immunotherapy by killing tumor targets *via* the secretion of various mediators including TNFα (33, 34). Thus, the functional activity of the macrophages identified as being MAC_inflam_ based on phenotypic markers was verified by their ability to lyse tumor targets (Figure 5). Consistent with earlier findings, R848-treated mMDSCs lysed tumor targets as did cells cultured with IFNγ or the combination of IL-6 plus TNFα (Figure 5).

RNA-Seq was used to identify the genes and regulatory networks critical to the differentiation of mMDSC to MAC_inflam_. Analysis focused on those genes whose expression was significantly increased by all three forms of stimulation: R848, TNFα plus IL-6, and IFNγ. 82% of the genes upregulated by the cytokine combination were also activated by R848 as opposed to 37% common genes between IFNγ and R848 (Figure 6A). IPA analysis revealed that a majority of the genes whose expression was increased by all three stimulants were linked *via* networks involving TNFα, NF-κB, and the STAT4 pathways. Inhibiting either NF-κB or STAT4 transcription factors significantly reduced the differentiation of human mMDSC into MAC_inflam_ (Figure 6D). While consistent with evidence that NF-κB influences the differentiation of human monocytes, these findings are at odds with studies in mice showing that NF-κB activation causes MDSC to accumulate at sites of inflammation (supporting the importance of evaluating the activity of human mMDSC) (35, 36). Less is known of the role of STAT4 in the differentiation of myeloid cells. Originally identified as a transcription factor supporting the maturation of Th1 and NK cells, it is expressed by activated blood monocytes (37, 38). Unfortunately, no STAT4-specific inhibitor has been described, so Ruxolitinib was used in this work to monitor inhibition. Ruxolitinib blocks signal transduction mediated by multiple STATs (39). As STAT4 was the only member of the STAT family significantly upregulated by R848, IFNγ, and TNFα plus IL-6, this combination of findings suggest that STAT4 plays a role in the differentiation of human mMDSC into MAC_inflam_. Importantly, one of the four targets jointly regulated by NF-κB and STAT4 was inhibitor of STAT3 (SOCS3, Figure 6C) (40). STAT3 has been implicated in the maintenance and function of MDSCs in cancer patients (41, 42). Our findings are consistent with the possibility that inflammatory stimuli drive mMDSC to differentiate into MAC_inflam_ by limiting STAT3 activity, a conclusion supportive of further development of STAT3 inhibitors for clinical use. However, the role of STAT3 was not analyzed in this study given that currently available inhibitors lack specificity and RNA interference is accompanied by technical challenges related to primary myeloid cultures (43, 44). By contrast, upregulation of IL-6 and IL-10 was present only when mMDSC were stimulated with R848 or IL-6 plus TNFα, while expression of multiple IRFs was found only in IFNγ stimulated cultures (Figures 6B,C and data not shown). These findings suggest that IL-6, IL-10, and IRFs can support but are not central to the generation of MAC_inflam_.

The therapeutic utility of R848 is limited by the development of lymphopenia (45, 46). Systemic administration of TNFα can lead to hypotension and hepatotoxicity while IL-6 is known to be present in the tumor microenvironment, where it supports the survival and proliferation of cancer cells (47, 48). Our findings indicate that the behavior of IL-6 is altered when combined with TNFα and that it augments TNFα-mediated conversion of mMDSC into tumoricidal MAC_inflam_ (Figure 5). Although IFNγ is a potent anti-tumoral agent, it is reported to support tumor growth by increasing the proliferative capacity of the cancer cells and upregulating immune checkpoint inhibitors *via* a negative feedback loop (49, 50). Current findings suggest that tumor growth might be inhibited by targeting the immunosuppressive milieu through local delivery of R848, IFNγ, or TNFα.

## Author Contributions

DB and DT performed the experiments and analyzed results; DB and DK designed the research, analyzed results, and wrote the paper; DK supervised the research.

## Conflict of Interest Statement

The authors declare that the research was conducted in the absence of any commercial or financial relationships that could be construed as a potential conflict of interest.

## Supplementary Material

Figure S1 Effect of longer incubation period on the percentage of MAC_inflam_ and MAC_suppress_. FACS-purified human monocytic myeloid-derived suppressor cells were stimulated with R848 (3 µg/ml) or PAM3 (1 µg/ml) for 3–5 days. The percentage of cells bearing MAC_inflam_ (25F9^+^, CD206^−^) versus MAC_suppress_ (25F9^+^, CD206^+^) phenotype is shown (mean ± SD of three independently studied donors per group). **p* < 0.05.
