# Supplementary figures and images for: Factors Influencing the Differentiation of Human Monocytic Myeloid-Derived Suppressor Cells Into Inflammatory Macrophages
Source: Front Immunol. 2018 Mar 26;9:608. doi: 10.3389/fimmu.2018.00608 (PMC5879147; doi:10.3389/fimmu.2018.00608)

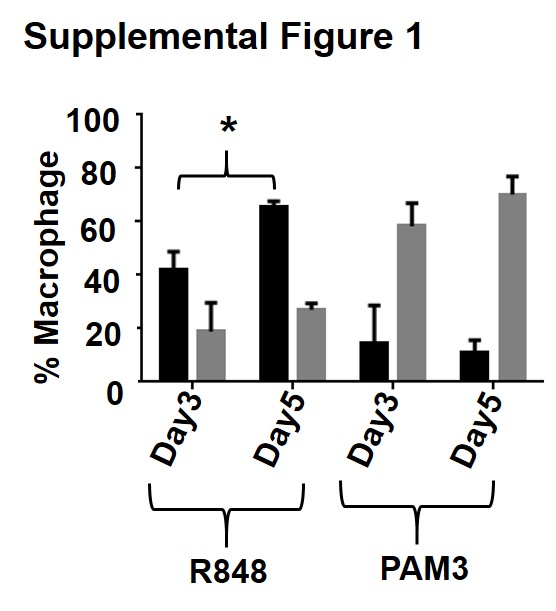

Supplement: Figure S1 — Effect of longer incubation period on the percentage of MACinflam and MACsuppress. FACS-purified human monocytic myeloid-derived suppressor cells were stimulated with R848 (3 µg/ml) or PAM3 (1 µg/ml) for 3–5 days. The percentage of cells bearing MACinflam (25F9+, CD206−) versus MACsuppress (25F9+, CD206+) phenotype is shown (mean ± SD of three independently studied donors per group). *p < 0.05. [file image_1.jpeg]
